# Comparative Analysis of Blood and Bone Marrow for the Detection of Circulating and Disseminated Tumor Cells and Their Prognostic and Predictive Value in Esophageal Cancer Patients

**DOI:** 10.3390/jcm9082674

**Published:** 2020-08-18

**Authors:** Florian Richter, Annette Baratay, Christian Röder, Jan-Hendrik Egberts, Holger Kalthoff, Thomas Becker, Susanne Sebens

**Affiliations:** 1 Department of General, Visceral-, Thoracic-, Transplantation- and Pediatric Surgery, University Medical Center Schleswig-Holstein (UKSH), Campus Kiel, 24105 Kiel, Germany; florian.richter@uksh.de (F.R.); Jan-Hendrik.Egberts@uksh.de (J.-H.E.); thomas.becker@uksh.de (T.B.); 2 Institute for Experimental Cancer Research, Kiel University (CAU) and University Medical Center Schleswig-Holstein (UKSH), Campus Kiel, 24105 Kiel, Germany; afolkers@web.de (A.B.); Christian.Roeder@uksh.de (C.R.); hkalthoff@email.uni-kiel.de (H.K.)

**Keywords:** esophageal cancer, disseminated tumor cells, circulating tumor cells, cytokeratin-20, biomarkers, targeted therapies

## Abstract

Hematogenic tumor cell spread is a key event in metastasis. However, the clinical significance of circulating tumor cells (CTC) in the blood and disseminated tumor cells (DTC) in bone marrow is still not fully understood. Here, the presence of DTC and CTC in esophageal cancer (EC) patients and its correlation with clinical parameters was investigated to evaluate the CTC/DTC prognostic value in EC. This study included 77 EC patients with complete surgical tumor resection. CTC and DTC were analyzed in blood and bone marrow using nested CK20 reverse transcription-nested polymerase chain reaction (RT-PCR) and findings were correlated with clinical data. Twenty-seven of 76 patients (36.5%) showed CK20 positivity in the blood, 19 of 61 patients (31.1%) in bone marrow, and 40 (51.9%) of 77 patients were positive in either blood or bone marrow or both. In multivariate analyses, only the DTC status emerged as independent predictor of overall and tumor specific survival. Our study revealed that, while the presence of CTC in blood is not associated with a worse prognosis, DTC detection in the bone marrow is a highly specific and independent prognostic marker in EC patients. Larger cohort studies could unravel how this finding can be translated into improved therapy management in EC.

## 1. Introduction

Esophageal cancer (EC) is still one of the most aggressive epithelial tumors and its incidence is increasing [1]. The mean 5-year survival rate is between 10–25%, making it one of the carcinomas with a particularly poor prognosis and a mortality rate of up to 90% [2], which can be partly explained by the fact that only one in seven tumors is detected at an early stage (T1) [3,4]. Pre-operative radiologic and endoscopic procedures are performed in order to detect visible metastases of esophageal carcinomas, and to perform a first therapy decision. In addition, pre-operative diagnostics serve to differentiate whether endoscopic removal of the tumor is possible, or whether surgical resection is necessary [5,6]. In case of a locally advanced tumor disease, a surgical esophago-lymphadenectomy is a major component of a multimodal therapy [7,8]. The preferred curative treatment of EC is transthoracic esophagectomy with 2-field lymphadenectomy and reconstruction by gastric elevation [9]. This procedure allows en-bloc resection of the esophagus and extensive mediastinal lymphadenectomy [10]. Then, post-operative tumor staging is assigned to UICC stages I-IV according to the TNM classification of the UICC. The TNM classification basically provides a good prognosis in terms of metastasis-free and overall survival, however, it still has an uncertainty in early tumor stages, despite continuous updates and adaptations to new diagnostic procedures. Especially in early tumor stages, a correct TNM classification is usually difficult, due to only minor cellular changes and unclear lymph node status. Moreover, it has been shown that even in 50% of patients, having undergone surgery and without any signs of lymph node or distant metastases at that time, a local recurrence or distant metastasis occurs within 12 months after surgery [11].

The most likely explanation for this can be seen in the already occurred detachment of tumor cells from the primary tumor, their spreading in the body and persistence as circulating tumor cells (CTC) in the blood or as disseminated tumor cells (DTC) in various organs or compartments of the body [12,13]. The process by which tumor cells evade their primary cellular environment and acquire migratory and invasive capacity is known as epithelial–mesenchymal transition (EMT), which has been described in many epithelial tumors [14,15].

During EMT, carcinoma cells lose epithelial characteristics comprising the loss or downregulation of epithelial marker proteins and concomitantly gain mesenchymal properties. For metastatic outgrowth at secondary sites, this process has to be reverted in order to allow the cell’s switch from a migratory to a proliferative stage, a process which is called mesenchymal–epithelial transition (MET). Notably, only those tumor cells which exhibit a high cellular plasticity and being able to change between those epithelial and mesenchymal states are thought to give rise to a detectable metastasis. Thus, CTC and DTC may exhibit different phenotypes with respect to epithelial and mesenchymal characteristics, which also implies a high functional heterogeneity (non-dividing versus proliferating, sessile versus motile) [16]. Since CTC and DTC are rare events (for solid tumors a number of 1 cell per 5–10 × 10^6^ white blood cells is assumed), they are not detected by current clinical imaging modalities, although metastatic spread may have already started much earlier before diagnosis of the (resectable) primary tumor [17]. Thus, the identification of markers and techniques that provide additional prognostic and predictive information, and that help to identify patients at risk for future metastases, are urgently needed [18].

In addition to the established TNM classification based on the excised tumor tissue, the concept of analyzing prognostic and predictive biomarkers in “liquid biopsies” has attracted great interest in recent years, because of the often non-invasive sample accessibility [19,20]. One focus of liquid biopsy analyses is on CTC in blood and DTC in bone marrow, which may add additional information relevant to the prognosis and contain molecular signatures of the tumor [21,22]. While this concept is well established in breast cancer patients, comprehensive analyses in EC patients are still lacking. However, CTC have been already identified in peripheral blood of EC patients and it could be shown that CTC are an independent prognostic parameter [23,24]. Furthermore, it has already been demonstrated that there is a correlation between the detection of DTC in the bone marrow of EC patients and an increased risk of metastases after successful surgery [25].

To date, various techniques for CTC enrichment and detection have been established and applied. The detection of these rare cells is principally performed either by a cellular or molecular approach [17,26,27]. However, during EMT, carcinoma cells may lose cell surface expression of typical epithelial proteins such as EpCAM, which is often used in cellular enrichment and detection approaches. This implies a considerable risk for an underestimation of the CTC count [28]. Therefore, in this study, a general cell-enrichment technique by centrifugation through Ficoll was used followed by a reverse transcription-nested polymerase chain reaction (RT-PCR) to detect cytokeratin-20 (CK20) mRNA as a marker for CTC in blood and DTC in bone marrow of EC patients. CK20 is a structural protein specific for epithelial cells of endo- and ectodermal origin, but exhibits no expression—with very limited exceptions [29]—in mesenchymal compartments, such as the vascular and the hematopoietic system.

Thus, even though the applied CK20 RT-PCR detects mRNA specific for epithelial cells in general rather than carcinoma specific mRNA, this approach has been identified to possess a very high sensitivity and specificity to detect these rare cells in different body compartments of cancer patients [30,31,32]. Despite the general availability of CK20-specific state-of-the-art real-time polymerase chain reaction (PCR) assays, which can be optimized towards very high sensitivity and specificity e.g., by using TaqMan technology in combination with optimized PCR primers [33], the nested endpoint PCR approach used in our analysis was optimized for very high input of target RNA/cDNA. This strongly improved the detection of the very rare CTC/DTC sequences in the tested analytes.

Thus, the aim of this study was the detection of CK20 mRNA expression in blood and bone marrow of a well characterized cohort of 77 EC patients as an indicator for the presence of CTC and DTC, and to assess its prognostic value. Furthermore, CK20 positivity was correlated with various clinical parameters, in order to investigate whether pre-operative CTC/DTC detection provides a more accurate prognostic measure for EC patients, and might therefore improve pre-operative staging.

## 2. Methods

### 2.1. Patient Cohort

In total, the patient collective of this retrospective single-center study consisted of 77 EC patients. All patients underwent complete surgical tumor resection (R0) in the Department of General Surgery and Thoracic Surgery, University Hospital Schleswig-Holstein (UKSH), Campus Kiel, and were histologically verified as esophageal carcinoma at the Institute of Pathology UKSH Campus Kiel between 1996 and 2006. The median follow-up period was 89 months (95% confidence interval: 81–98). All patients were excluded who were lost to follow-up within the study period (1 patient) or who had died peri-operatively (up to 4 weeks after surgery) (3 patients). In addition, 9 patients were excluded, because no information was available regarding whether they had received neo-adjuvant chemotherapy. Patients with squamous cell carcinoma and adenocarcinoma were included. In the group of patients with adenocarcinomas of the esophagogastric junction (AEG) only type I and II were included. Patients with an AEG III tumor were not included, as they were classified as gastric carcinoma, according to the current classification [34].

The study was approved by the local ethics committee of the UKSH Campus Kiel and the Medical Faculty, Kiel University (reference no. A110/99). All patients gave written informed consent before inclusion in the study. Classification of the pathological tumor stage was conducted by the Department of Pathology, UKSH Campus Kiel according to the TNM-classification 7th Edition. Patient samples were processed and stored by the oncological biobank BMB-CCC. Clinical data were extracted from patient’s files and stored in a clinical research database of the oncological biobank BMB-CCC. Follow-up data were obtained from the Cancer Registry Schleswig-Holstein (Bad Segeberg, Germany). Clinical and follow-up data were then analyzed in correlation to the CK20 expression detected by nested RT-PCR. Expression data were used to stratify patients at risk, and the prognostic relevance of CK20 expression in blood and bone marrow samples of EC patients was analyzed.

### 2.2. Liquid Biopsy Collection

Immediately prior to surgery, a blood sample was drawn from a central venous line into a lithium heparin monovette (Sarstedt, Nümbrecht, Germany). The bone marrow was punctured at the right iliac crest and 10 mL of bone marrow blood were taken and drawn into a lithium heparin monovette as well. All samples were kept at room temperature (18–25 °C) and were further processed within 0.5–2 h. Separation of the mononuclear cell fraction was performed by centrifugation through a Ficoll-Hypaque density cushion (GE Healthcare, Freiburg, Germany). Mononuclear cell fractions were then isolated, washed in phosphate buffer saline (PBS) and cells were counted in a Neubauer chamber. For RNA preparation, cells were lyzed in PeqGold RNApure™ reagent (PeqLab, Erlangen, Germany) and total RNA was isolated according to the manufacturer’s protocol.

### 2.3. Nested RT-PCR and Analysis

Total RNA (2.0 µg) in a volume of 10 µL was denatured for 10 min at 70 °C and quickly chilled on ice. CDNA was synthesized in a total volume of 20 µL containing 5 × first-strand buffer, 2 mM DTT, 200 units of SuperScript II (all from Life Technologies, Karlsruhe, Germany), 4 units of RNase inhibitor, 2.5 µM random hexamer, 0.5 mM of each deoxynucleotide triphosphate mixture (Perkin-Elmer Corp., Weiterstadt, Germany). Incubation for 10 min at 24 °C and 60 min at 42 °C was followed by an inactivation step for 5 min at 95 °C. Primers were synthesized by Eurofins Genomics, Ebersberg, Germany as follows: CK20-222 (sense), 5′-GCGTTTATGGGGGTGCTGGAG; CK20-988 (antisense), 5′-AAGGCTCTGGGAGGTGCGTCTC; CK20-304 (sense), 5′-CGGCGGGGACCTGTTTGT; CK20-767 (antisense), 5′-CAGTGTTGCCCAGATGCTTGTG; PBGD (sense), 5′-CTTCACCATCGGAGCCATCTGC; PBGD (antisense), 5′-CGAAGCCGGGTGTTGAGGTTT. PBGD (porphobilinogen deaminase)-cDNA was amplified as a housekeeping probe to verify RNA/cDNA quality for PCR.

For the first round of (external) PCR reaction, 30 µL of a PCR mixture containing 1 × Tricine buffer, 200 µM deoxynucleotide triphosphate mixture (dNTP), CK20-222 and CK20-988 primers (0.4 µM each), and 1 unit of Taq DNA polymerase (Life Technologies, Karlsruhe, Germany) was added to the cDNA preparation (final volume: 50 µL). 10 × Tricine buffer [35] contained 300 mM tricine, pH 8.4, 20 mM MgCl_2_, 50 mM 2-mercaptoethanol, 0.1% gelatin, 1% thesit. The cycling protocol is indicated in Table 1. The second, nested PCR was performed with a 1 µL external PCR reaction as the target, using the same cycling protocol, tricine-PCR mixture, 200 µM dNTP, nested primer pairs CK20-304 and CK20-767 (0.4 µM each) and 1 unit Taq polymerase in a 50 µL volume. RT-PCR products were separated by electrophoresis in a 2% agarose gel (MetaPhor agarose; Biozym, Hessisch Oldendorf, Germany) in Tris-acetate-EDTA buffer and visualized by ethidium bromide staining. The molecular weights were determined using a DNA molecular weight standard (DNA marker VIII, Roche Diagnostics, Mannheim, Germany).

The nested CK20 PCR yielded a 485 bp product and the PBGD product had a length of 204 bp. For the CK20 nested RT-PCR, the samples were tested twice. If the 485 bp CK20 amplicon was detected in at least one PCR run, the sample was judged as positive. Samples without detectable PCR-product were considered as negative. The high sensitivity and specificity of this CK20 nested RT-PCR assay was validated as demonstrated in our earlier reports [32,36], using peripheral blood and bone marrow samples from 38 control patients, and blood samples from 38 healthy donors. Among the control cases were 24 patients with nonmalignant diseases (liver cysts, liver adenoma, diverticulitis, familial adenomatous polyposis, pancreatitis, hernias, ulcera ventriculi, primary sclerosing cholangitis), 6 bone marrow donors and 8 leukemia patients. In bone marrow and venous blood samples of this clinical control group, we observed CK20 positivity in 3 patients, all with premalignant diseases. One patient with an extended liver adenoma tested positive in both, blood and bone marrow. A patient with familial adenomatous polyposis was positive in the bone marrow, and a patient with chronic pancreatitis showed CK20 positivity in the blood. All other samples, including those from 38 healthy control subjects, were negative for CK20.

The CK20 nested RT-PCR assay was additionally validated earlier testing total RNA from several human gastrointestinal cancer cell lines as positive controls and investigating serial dilutions of these samples [31]. In this report, by utilizing serially diluted samples, the superior sensitivity of the nested PCR approach in comparison to a single 30-cycle PCR could be clearly demonstrated.

### 2.4. Statistical Analysis

Statistical analyses were performed for all subsets of clinical parameters combined, and then independently for tumor site and histopathological staging. Kaplan–Meier survival analyses were carried out for overall and disease-free survival (OS, DFS). For univariate analysis, the statistical significance was assessed by the log rank test. The correlation between the detection rate of CK20 and clinical parameters was analyzed with the χ2 test after crosstab examination. Variables showing a significant association with the detection of a biomarker in univariate analysis were included in multivariate models. Cox proportional hazard models were used in multivariate analysis.

All reported *p*-values are two-sided and were regarded statistically significant at ≤0.05. Statistical calculation and testing was performed with IBM SPSS Statistics 23.0 (IBM, München, Germany).

## 3. Results

### 3.1. Characterization of the Analyzed Patient Cohort

The patient collective consisted of 77 patients with a median age of 61 years. Out of the 77 patients, 64 (83.1%) were male and 13 (16.9%) female. 37(48.1%) patients presented with squamous cell carcinoma, 39 (50.6%) with adenocarcinoma (1.3%) and one patient with a neuroendocrine tumor. Furthermore, 32 (41.6%) of the patients had received neo-adjuvant chemotherapy, whereas 45 (58.4%) of the patients were untreated before surgery. All data are shown in Table 2. The observed recurrence rate in the present patient population was 46.75% during the observation period. The median survival in the patient collective was 26 months. The 5-year overall survival was 33.23%.

### 3.2. Detection Rates of CK20 mRNA by RT-PCR

**Figure 1:**
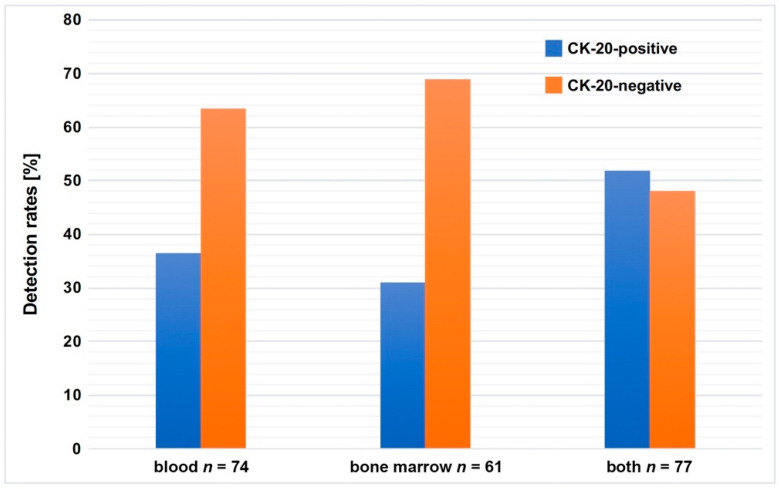
Detection rates of CK20 mRNA in blood or bone marrow or blood and bone marrow of patients with esophageal carcinoma. Blood samples from 74 esophageal cancer (EC) patients and bone marrow samples from 61 EC patients were analyzed for the presence of CK20 mRNA. Data are presented as % detection rate.

In order to investigate whether CK20 expression, a known surrogate marker for the presence of CTCs in patients with colorectal cancer [26], can also be detected in blood and bone marrow samples of EC patients, a nested CK20 RT-PCR was performed with cDNA prepared from 74 blood and 61 bone marrow samples of 77 EC patients. As shown in Figure 1, 27 of 74 (36.5%) blood samples tested positive for CK20 mRNA and, in 19 of 61 (31.1%) bone marrow samples, CK20 expression could also be detected. Furthermore, it was shown that 40 of the 77 patients (51.9%) presented positive evidence for CK20 mRNA in both body compartments.

### 3.3. Correlation of CK20 mRNA Detection Levels in Blood and Bone Marrow and Patient’s Survival

**Figure 2:**
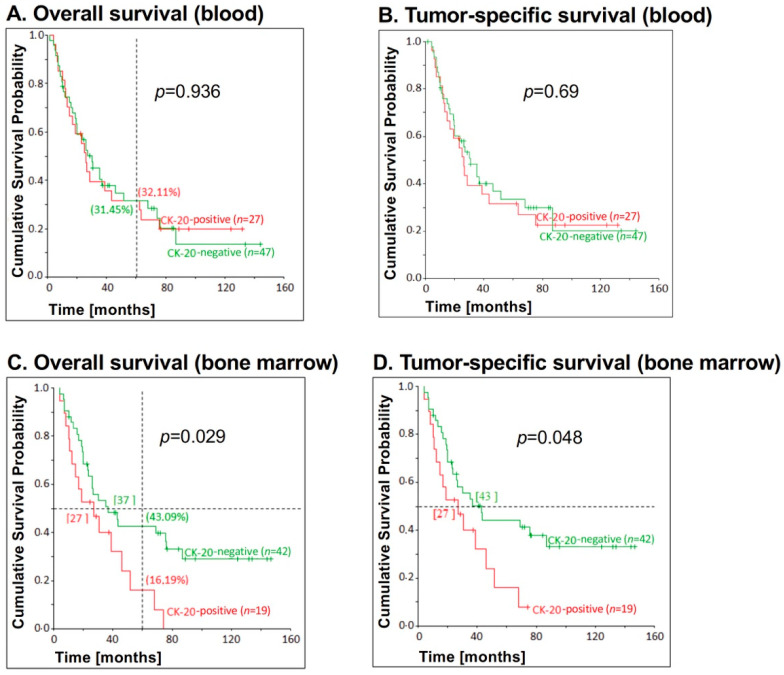
Correlation between CK20 positivity in blood and bone marrow samples, respectively, and survival for patients with esophageal carcinoma. CK20 positivity in (**A**,**B**) blood (*n* = 27) and (**C**,**D**) bone marrow (*n* = 19) samples of EC patients was assessed by reverse transcription-nested polymerase chain reaction (RT-PCR) and correlated with (**A**,**C**) overall survival and (**B**,**D**) tumor-specific survival. On the vertical dotted line at 60 months, the percentages of 5-year overall survival are shown in brackets. The *p*-values for the comparison of the cumulative survival probabilities of the two groups (CK20-positive versus CK20-negative) were calculated using the log-rank test. The horizontal dotted line at the cumulative survival probability of 0.5 gives the median survival and is given in months in square brackets.

Next, we analyzed whether CK20 mRNA detection correlates with survival of EC patients. As shown in Figure 2, no significant correlation between CK20 positivity in blood samples and overall survival (*p* = 0.936) (Figure 2A) or tumor-specific survival (*p* = 0.69) (Figure 2B) could be determined.

However, there was a significant correlation between bone marrow CK20 positivity and overall survival (*p* = 0.029), as well as tumor-specific survival (*p* = 0.048). After five years, 43.09% of the CK20-negative patients, but only 16.19% of the CK20-positive patients were still alive (Figure 2C). Moreover, half of the patients with CK20-negative bone marrow samples had died after 37 months (43 months: tumor-specific survival), while half of the patients with CK20-positive samples had already died after 27 months (also tumor-specific survival) (Figure 2C,D).

### 3.4. Correlation between CK20 mRNA Detection in Blood and Bone Marrow and Survival of EC Patients in Dependence on UICC Stage

**Figure 3:**
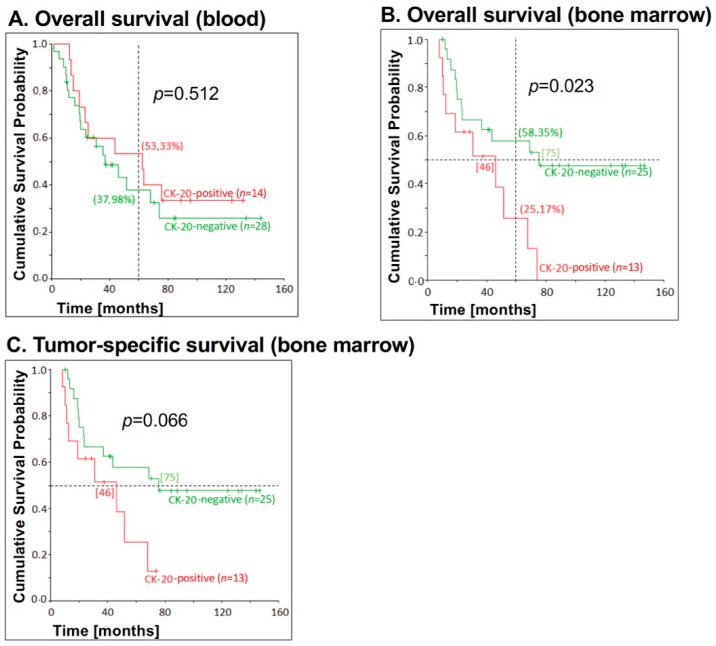
Correlation between CK20 positivity in blood and bone marrow samples, respectively, and survival for patients with esophageal carcinoma UICC stage I and II. CK20 positivity in (**A**) blood (*n* = 20) and (**B**,**C**) bone marrow (*n* = 13) samples of EC patients with lower tumor stages were assessed by RT-PCR and correlated with (**A**) overall survival in blood samples, (**B**) overall survival and (**C**) tumor-specific survival in bone marrow samples. The *p*-values for the comparison of the cumulative survival probabilities of the two groups (CK20-positive versus CK20-negative) were calculated using the log-rank test. On the vertical dotted line at 60 months, the percentages for 5-year overall survival are shown in brackets. On the horizontal dotted line at the cumulative survival probability of 0.5 is the median survival and is given in months in square brackets.

Next, we analyzed whether the correlation of CK20 expression in blood and bone marrow samples of EC patients and survival is dependent on the UICC stage. The association between blood CK20 mRNA detection and survival for patients with UICC stages I and II was not significant for overall survival (*p* = 0.512), recurrence-free survival (*p* = 0.582) and tumor-specific survival (*p* = 0.597) (Figure 3A). Deducing from the Kaplan–Meier curve shown for overall survival, patients with CK20-positive blood in early UICC stages paradoxically had a significantly higher 5-year survival than patients with CK20-negative samples (53.33% versus 37.98%) (Figure 3A).

Out of the 44 patients in UICC stages I or II, bone marrow samples could be tested of 36 patients, of whom 13 (36.11%) were CK20-positive. Blood samples were tested from 42 patients, of whom 14 (33.33%) were positive.

While no significant correlation between CK20 mRNA detection in blood samples and overall survival (*p* = 0.261) or tumor-specific survival (*p* = 0.196) could be determined (Figure 3A), a significant correlation between bone marrow CK20 positivity and overall survival for EC patients with UICC stages I and II (*p* = 0.023) could be observed (Figure 3B,C). Thus, patients lacking CK20 expression in the bone marrow showed a significantly longer survival than patients with CK20-positive bone marrow (Figure 3B). The correlation between CK20 bone marrow positivity and tumor-specific survival (*p* = 0.066) was not significant in patients with UICC stage I and II, but there was a clear trend towards different courses of the disease, as shown in the Kaplan–Meier graph. While CK20-negative patients had a median survival of 75 months for both overall and tumor-specific survival, CK20-positive patients only had a median survival of 46 months for overall and tumor-specific survival (Figure 3B,C).

Of the 29 patients with UICC stage III or IV, bone marrow samples of 23 patients were tested, six (26.09%) of which were positive. Blood samples were tested for 28 patients, of whom 12 (42.86%) were CK20-positive.

**Figure 4:**
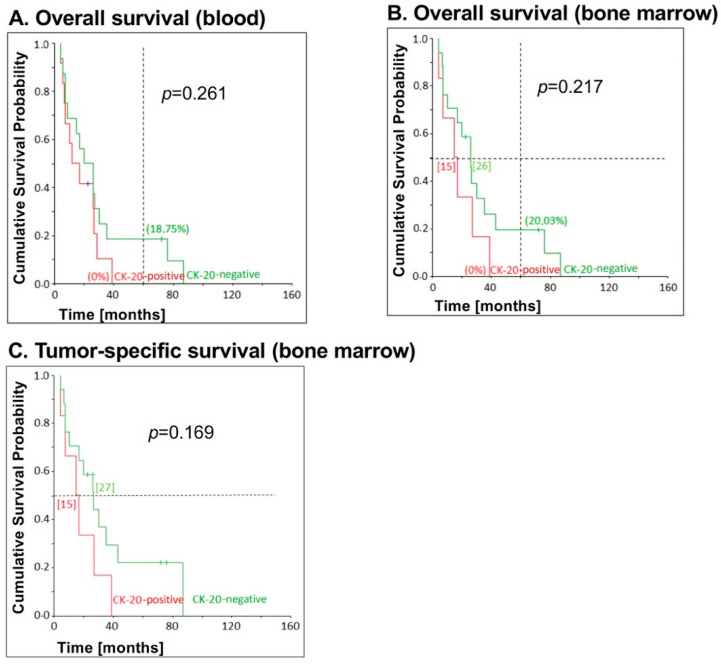
Correlation between CK20 positivity in blood and bone marrow samples, respectively, and survival for patients with esophageal carcinoma UICC stage III and IV. CK20 positivity in (**A**) blood (*n* = 12) and (**B**,**C**) bone marrow (*n* = 6) samples of EC patients with advanced tumor stages was assessed by RT-PCR and correlated with (**A**) overall survival in blood samples, (**B**) overall survival und (**C**) tumor-specific survival in bone marrow samples. The *p*-values for the comparison of the cumulative survival probabilities of the two groups (CK20-positive versus CK20-negative) were calculated using the log-rank test. On the vertical dotted line at 60 months, the percentage 5-year overall survival are shown in brackets. On the horizontal dotted line at the cumulative survival probability of 0.5 is the median survival and is given in months in square brackets.

While no significant correlation between CK20 mRNA detection in blood and overall survival (*p* = 0.261) or tumor-specific survival (*p* = 0.196) could be determined in patients with early UICC stages, patients with late UICC stages (UICC III+IV) showed a higher 5-year overall survival when blood samples were tested CK20-negative than CK20-positive patients (Figure 4A). Thus, 18.75% of the CK20-negative patients were still alive after 5 years, while all of the CK20-positive patients had already died after 39 months (Figure 4A).

There was no significant correlation between bone marrow CK20 positivity and overall survival (*p* = 0.217) and tumor-specific survival (*p* = 0.169) (Figure 4B,C). However, the percentage of CK20-negative patients being alive after 5 years was significantly higher than that of patients with a CK20-positive bone marrow. Thus, regarding overall survival, 20.03% of the patients with CK20-negative bone marrow samples were still alive after 5 years, whereas all patients with CK20-positive samples had already died after 39 months. The median survival of CK20-negative patients was also longer. Of these, half of the patients were still alive after 26 months (tumor-specific: 27 months), while half of the patients with CK20-positive samples had already died after 15 months (also tumor-specific). Furthermore, half of the patients with CK20-negative bone marrow had relapsed or died after 17 months, while in the CK20-positive patients this had already occurred after 15 months (Figure 4C).

### 3.5. Correlation between CK20 mRNA Detection in Blood and Bone Marrow and Survival in EC Patients without Neo-Adjuvant Chemotherapy

To investigate whether the expression of CK20 has an influence on survival of patients without pre-operative therapy, 43 blood and 36 bone marrow samples from 45 EC patients who did not receive neo-adjuvant therapy were examined.

In these patients, no significant correlation between CK20 mRNA detection in blood and overall survival (*p* = 0.776) as well as CK20 positivity in blood and tumor-specific survival (*p* = 0.593) could be assessed (data not shown).

**Figure 5:**
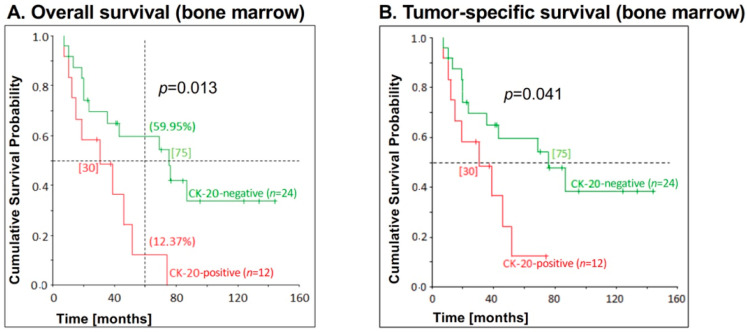
Correlation between CK20 positivity in bone marrow samples and survival for patients with esophageal carcinoma who had not received neo-adjuvant chemotherapy. CK20 positivity in bone marrow of EC patients who had not received neo-adjuvant chemotherapy (*n* = 12) was assessed by RT-PCR and correlated with (**A**) overall survival and (**B**) tumor-specific survival. The *p*-values for the comparison of the cumulative survival probabilities of the two groups (CK20-positive versus CK20-negative) were calculated using the log-rank test. On the vertical dotted line at 60 months, the percentages of 5-year overall survival are shown in brackets. On the horizontal dotted line at the cumulative survival probability of 0.5 is the median survival and is given in months in square brackets.

Furthermore, there was a significant association between bone marrow CK20 positivity and overall survival (*p* = 0.013), as well as bone marrow CK20 positivity and tumor-specific survival (*p* = 0.041) in this patient cohort (Figure 5). Thus, in contrast to the overall collective, there was also a significant correlation between CK20 mRNA detection in bone marrow and recurrence-free survival in EC patients who did not undergo neo-adjuvant therapy. EC patients with a CK20-negative bone marrow had a significantly higher survival than CK20-positive patients. The 5-year overall survival for CK20-negative patients was 59.95% compared to 12.37% for patients with a CK20-positive bone marrow. Of the CK20-positive patients in this collective, half of the patients had already died after 30 months (also tumor-specific), in comparison to 75 months (also tumor-specific) for CK20-negative patients. After 24 months, half of all of CK20-positive patients had already relapsed or died in contrast to patients with CK20-negative bone marrow who relapsed after 73 months (Figure 5).

### 3.6. Detection Rates of CK20 mRNA in Liquid Biopsies of EC Patients Associated with Clinical Parameters

Next, we analyzed whether the detection of CK20 expression in liquid biopsies of EC patients is correlated with distinct clinical parameters. In our collective, neo-adjuvant therapy did not influence the detection of CK20 rate in blood samples (*p* = 0.736). The recurrence rate was also not associated with the detection of CK20 mRNA in blood (*p* = 0.209). There was a significantly higher detection rate of CK20 mRNA in blood of patients with squamous cell carcinomas than with adenocarcinomas (45.71% versus 26.32%); however, this correlation was not significant (*p* = 0.084) (Table 3).

Examination of bone marrow samples revealed no correlation between CK20 positivity and any of the clinical parameters tested. Although 32.69% male patients and only 22.22% of female patients had a CK20-positive bone marrow, the Chi-square test revealed no correlation between CK20 mRNA detection in bone marrow and sex (*p* = 0.531).

As observed for blood samples, the detection rate of CK20 mRNA in bone marrow was slightly lower by trend in patients with adenocarcinomas (detection rate 24.14%), than in those with squamous cell carcinomas (detection rate 35.48%) (Table 4).

The following correlations between CK20 positivity and clinical parameters could be shown in the patient collective consisting of blood and/or bone marrow. The detection rate of CK20 mRNA in blood and/or bone marrow was dependent on the UICC stage (*p* = 0.038). Seven of 16 patients (43.75%) with stage I showed a CK20-positive sample, while in 28 patients with stage II, the CK20 mRNA detection rate was, with 64.29%, significantly higher, and was highest in UICC stage IV patients. Here, 8 of 10 patients (80%) showed CK20 positivity in blood and/or bone marrow. In contrast, only 16 of the 32 patients (50%) with stage III UICC were CK20-positive in either compartment.

Patients with distant metastases (M1) had significantly higher detection rates of CK20 mRNA in blood and/or bone marrow than patients without distant metastases (80% versus 48.48%). No correlation between the detection of CK20 mRNA in blood and/or bone marrow and treatment with a neo-adjuvant therapy could be demonstrated (*p* = 0.891). Furthermore, the recurrence frequency did not depend on the CK20 mRNA detection rate in blood and/or bone marrow (*p* = 0.891). When analyzing the histological tumor type, it was noticeable that the CK20 mRNA detection rate for patients with adenocarcinomas was lower than for patients with squamous cell carcinomas (41% versus 62.16%, *p* = 0.065) (Table 5).

### 3.7. Results of the Multivariate Analyses

In order to investigate whether detection of CK20 expression in liquid biopsies is an independent prognostic factor for EC patients, multivariate analyses using the Cox regression model were performed and calculated, using the stepwise inclusion method and the likelihood quotient (LQ) method. Only variables with a significant *p*-value in the univariate analysis (log-rank test) were included in the calculation. Thus, CK20 positivity in bone marrow was included in the calculations for overall and tumor-specific survival, whereas CK20 mRNA detection in blood samples was not. By multivariate analyses, detection of DTC in the bone marrow by CK20 RT-PCR was identified as an independent predictor of worse overall survival (HR 2.53; *p*  =  0.006). A higher UICC stage was also determined as an independent marker of worse overall survival (stage I and II versus stage III and IV, HR = 3.128, *p* < 0.001) and relapse free survival (stage I and II versus stage III and IV, HR = 2.91, *p* = 0.001). Finally, these markers were also independent predictors of a worse tumor-specific survival of EC patients (Table 6).

## 4. Discussion

The aim of this study was to investigate the prognostic and predictive value of CK20 mRNA expression level in blood and bone marrow in resectable EC patients. For this purpose, only patients with no residual tumor after surgery of esophageal cancer (R0 status) were examined. Even though CK20 is not an exclusive marker for tumor cells, because it is expressed by any epithelial cell of endo- and ectodermal origin, this marker has been already proven a robust indicator for CTC and DTC in colorectal carcinoma patients with a high prognostic and predictive value [26,37]. Since there are only a few studies that have analyzed blood and bone marrow in EC patients for the presence of CTC and DTC [38], the present study is the first one analyzing the presence of both CTC and DTC in a cohort of 77 EC patients.

We used a highly sensitive and specific nested CK20 RT-PCR subsequent to a Ficoll-based enrichment of the PBMC-fraction also containing circulating epithelial cells from blood and bone-marrow to detect CTC and DTC. With this technique, we were able to achieve detection rates of 36.5% in the blood and 31.1% in the bone marrow. This technique is validly more sensitive than surface antigen-based enrichment procedures, which yielded detection rates of 2 to 25.6% for CTC, and up to 17.1% for DTC [38,39,40,41].

The major limitation of immunomagnetic enumeration methods (e.g., using EpCAM as a surface marker) is that only a subset of CTC expressing this marker is detected, because it has been shown that CTC may have undergone EMT, by which the cells become motile, disseminate in the body and downregulate EpCAM expression [42,43]. Thus, strategies combining the analysis of more than one epithelial marker (e.g., cytokeratin and EpCAM) have been established providing a more reliable detection of CTC and DTC thereby increasing the detection rates also in EC patients [41]. Importantly, CTC and DTC, which have undergone EMT and exhibit a more mesenchymal phenotype, might be a cellular subgroup of high clinical relevance, because these cells seem to be characterized by a particularly aggressive metastatic potential along with drug resistance [44]. Accordingly, the difficulty of capturing this aggressive subgroup of cells by immunomagnetic approaches might also explain the results showing that detection of CTC and DTC did not correlate with survival and prognosis of cancer patients [45].

Interestingly, the detection of CTC in our cohort of EC patients did not correlate with the UICC stage, TNM category, gender, histological tumor type, recurrence rate or frequency of neo-adjuvant chemotherapy. However, we could observe a relatively high detection rate of CK20 expression in the blood of stage IV EC patients. Out of 10 patients with distant metastases, 60% (6/10) were positive for CK20 in the blood, while only 33.3% (21/63) of 63 patients without distant metastases were tested positive for CK20 mRNA. Contrary to the available literature, no significant correlation of the CTC count in the blood and overall survival, recurrence-free survival and tumor-specific survival could be shown [46,47,48,49,50]. Thus, further studies with larger EC patient cohorts are necessary to clarify the clinical importance of CTC in these cancer patients.

Similarly, no significant relation could be shown between CK20 positivity in bone marrow and the UICC stage, TNM category, gender, histological tumor type, recurrence rate or frequency of neo-adjuvant chemotherapy, which is in line with other studies demonstrating no correlation of DTC presence in bone marrow and clinical parameters in EC patients [51,52].

In contrast, a significant correlation between CK20 positivity in the bone marrow of EC patients and their survival could be shown. This applies to overall survival as well as tumor-specific survival. Moreover, there was also a correlation by trend between the presence of DTC in bone marrow and recurrence-free survival. Thus, after five years, only 16.19% of the patients with a CK20-positive bone marrow were still alive, whereas 43.09% of the patients being CK20-negative were alive. Furthermore, the multivariate Cox regression analysis revealed that the presence of CK20 mRNA expression in the bone marrow is an independent prognostic factor for overall survival and tumor-specific survival of EC patients. Such a negative correlation of DTC load in the bone marrow and survival has already been demonstrated for patients with different tumor diseases, such as EC, breast cancer, lung cancer, colorectal and pancreatic carcinoma [13,53,54,55,56].

Analysis of a sub-collective of 34 patients without lymph node metastases revealed a significant correlation between bone marrow CK20 positivity and overall survival, as well as tumor-specific survival, which was similar to the overall collective. For the sub-population of 44 patients with UICC stages I or II, there was also a significant correlation between CK20 mRNA detection in bone marrow and overall survival. However, no significant correlation could be demonstrated in patients with UICC stages III and IV. Similar results were reported by Bidard et al. [57]. A possible explanation for this is the more frequent treatment of patients with UICC stage III onwards with neo-adjuvant therapy. In our patient collective exhibiting CK20 positivity in the bone marrow, only 11 (39.29%) of the 28 stage II patients were treated with neo-adjuvant therapy, while nine (47.37%) of the 19 stage III patients had received neo-adjuvant chemotherapy.

Overall, these data indicate that an early UICC stage and a detection of DTC in bone marrow are of prognostic value for EC patients. Since patients with early UICC stages and lacking lymph node metastases do generally not receive neo-adjuvant therapy, the significant correlation between the detection of DTC in bone marrow and survival is particularly interesting and of clinical importance [58]. Another report showed that a considerable number of patients with the pre-operative tumor stage cT2-T3N0M0 presented a pN + status after therapy completion. Moreover, patients with this post-operative status showed a significantly worse prognosis than patients who had received neo-adjuvant treatment. Therefore, the majority of patients with a cT2-T3N0M0 stage should be considered for neo-adjuvant protocols and be treated by transthoracic resection whenever possible [59]. The work of van Hagen et al. highlighted impressively the significance of a pre-operative therapy, and clearly showed that neo-adjuvant chemotherapy offers a survival advantage over surgery alone [60].

These findings coincide with our data, which demonstrate a significant correlation between CK20 positivity in the bone marrow and neo-adjuvant therapy. By examining the sub-collective of 49 patients who had not received neo-adjuvant chemotherapy, it was shown that these patients were more often CK20-positive, which correlated with a shorter overall and tumor-specific survival. This may probably be explained by a partial elimination of DTC in the bone marrow by neo-adjuvant chemotherapy, as was shown previously [61]. However, there are also studies suggesting that DTCs are resistant to current therapeutic concepts and therefore persist in the bone marrow, even after chemotherapy [55]. Since patients who still harbor DTC in their bone marrow after chemotherapy have a particularly poor prognosis [61], these patients need to be identified at an early stage in order to optimize the therapeutic concept. Detecting DTC in bone morrow might provide a feasible strategy for identifying those patients who would benefit from neo-adjuvant or adjuvant therapy. Finally, as already suggested, e.g., for breast cancer patients, monitoring the DTC or CTC count in EC patients during and after therapy may help to monitor therapy responses and identify relapses much earlier than by conventional imaging modalities [62,63].

Concerning the correlation between TNM status and survival across the entire collective, our study demonstrated a significant correlation only between the N-category and the overall survival, which is identical to the literature [47,48]. However, patients without lymph node metastases showed a significantly longer overall survival than patients with lymph node metastases, which is in line with other studies [64].

Based on our study, we conclude that the presence of CTC in the blood detected by CK20 RT-PCR is only a snapshot without any prognostic value, while the detection of DTC in bone marrow clearly indicates a negative effect on long-term survival of EC patients.

Besides the prognostic value, a more precise characterization of CTC and DTC will help to better understand their biology providing the basis for a more reliable detection on the one hand and the development of distinct targeted therapies on the other hand. Therefore, a deepened knowledge on the cell’s plasticity as well as the conditions determining the different cellular stages [16,65,66] is required, which will allow the identification of novel markers and thereby enable a broader detection and improved targeting of CTC and DTC, respectively.

The recently published study by Sicklick et al. demonstrated that treatment with an individualized targeted therapy has a significant impact on survival of cancer patients [67]. A similar significance was given to targeted therapy in the article by Janmaat et al. [68]. Here, an individualized targeted therapy was even suggested as a standard therapy in case of a palliative situation in EC patients. Patients showing disease progression upon standard therapies should be subjected to genomic profiling and considered for clinical trials aimed at testing targeted therapies. Furthermore, it was shown in a recent report that Pembrolizumab can be used as a treatment option for PD-L1-positive patients with an already advanced tumor stage and Trastuzumab has been approved as first-line treatment in combination with chemotherapy for HER2/neu-positive patients [69]. Future targeted therapies may include CDK4/6 inhibitors, PARP inhibitors and inhibitors targeting the NRF2 and Wnt signaling pathways [70]. Combining research approaches of targeted therapies and early detection of recurrence via CTC and DTC assessment in liquid biopsies may help to improve the therapy, and therefore the recurrence-free survival of these patients.

In clinical oncology, there is an acute need for reliable biomarkers for real-time monitoring of the success of systemic adjuvant therapy in individual patients and the detection and enumeration of CTC/DTC has the potential to fill this important gap in oncology.

## 5. Conclusions

Our study revealed that, while the pre-operative presence of CTC in blood of EC patients is not associated with a negative prognostic influence, detection of DTC in the bone marrow by CK20 RT-PCR is highly specific, and an independent prognostic marker in EC patients. Future studies on a larger cohort have to unravel how this can be translated into an improved therapy management of these patients.

## Figures and Tables

**Table 1:** Cycling protocol for the nested CK20 and the PBGD polymerase chain reaction (PCR)**.**

Cycle	Denaturation	Primer Hybridization ^1^	Elongation	Cycles
Touch-down-cycles				
1	40 s/94 °C	2 min/70 °C		1
2	40 s/94 °C	1 min 55 s/69 °C		1
3	40 s/94 °C	1 min 50 s/68 °C (66 °C)		1
4	40 s/94 °C	1 min 45 s/67 °C (65 °C)		1
5	40 s/94 °C	1 min 40 s/66 °C (64 °C)		1
6	40 s/94 °C	1 min 35 s/65 °C (63 °C)		1
7	40 s/94 °C	1 min 30 s/64 °C (62 °C)		1
8	40 s/94 °C	1 min 25 s/63 °C (61 °C)		1
9	40 s/94 °C	1 min 20 s/62 °C (60 °C)		1
External and internal PCR				
10–30 31	40 s/94 °C	1 min/61 °C (59 °C)	1 min 30 s/72 °C 15 min/72 °C	21 1

^1^: temperatures in parentheses were used for the PBGD PCR.

**Table 2:** Characteristics of the analyzed cohort of patients with esophageal carcinoma.

	*n* (%)
All Sex	77 (100)
male	64 (83.1)
female	13 (16.9)
Tumor site	
Squamous cell carcinoma	37 (48.1)
adenocarcinoma	39 (50.6)
Neuroendocrine tumor	1 (1.3)
Neo-adjuvant treatment	
yes	32 (41.6)
no	45 (58.4)
Adjuvant treatment	
yes	2 (2.5)
no	53 (68.8)
unknown	22 (28.6)

**Table 3:** Correlation between CK20 mRNA detection in blood of esophageal carcinoma patients and clinical parameters.

Variable		Detection of CK20 mRNA in Blood (*n* = 74)			Correlation χ^2^-Test (*p*-Value)
		Total	Positive (%)	Negative (%)	
Gender	Female	13	7 (53.85%)	6 (46.15%)	0.152
	Male	61	20 (32.79%)	41 (67.21%)	
UICC-stage (*n* = 70)	I	16	5 (31.25%)	11 (68.75%)	0.447
	II	26	9 (34.62%)	17 (65.38%)	
	III	18	6 (33.33%)	12 (66.67%)	
	IV	10	6 (60%)	4 (40%)	
T-category (*n* = 71)	I	18	5 (27.78%)	13 (72.22%)	0.439
	II	22	7 (31.82%)	15 (68.18%)	
	III	30	14 (46.67%)	16 (53.33%)	
	IV	1	0	1 (100%)	
N-category (*n* = 74)	0	33	13 (39.39%)	20 (60.61%)	0.641
	I	41	14 (34.15%)	27 (65.85%)	
M-category (*n* = 73)	0	63	21 (33.33%)	42 (66.67%)	0.105
	I	10	6 (60%)	4 (40%)	
Neo-adjuvant therapy	Yes	31	12 (38.71%)	19 (61.29%)	0.736
	No	43	15 (34.88%)	28 (65.12%)	
Relapse	Yes	34	15 (44.12%)	19 (55.88%)	0.209
	No	40	12 (30%)	28 (70%)	
Tumor type (histological) (*n* = 73)	Adenocarcinoma SCC	38	10 (26.32%)	28 (73.68%)	0.084
		35	16 (45.71%)	19 (54.29%)	

**Table 4:** Correlation between CK20 mRNA detection in bone marrow of esophageal carcinoma patients and clinical parameters.

Variable		Detection of CK20 mRNA in Bone Marrow (*n* = 61)			Correlation χ^2^-Test (*p*-Value)
		Total	Positive (%)	Negative (%)	
Gender	Female	9	2 (22.22%)	7 (77.78%)	0.53
	Male	52	17 (32.69%)	35 (67.31%)	
UICC-stage (*n* = 70)	I	12	3 (25%)	9 (75%)	0.49
	II	24	10 (41.67%)	14 (58.33%)	
	III	15	3 (20%)	12 (80%)	
	IV	8	3 (37.5%)	5 (62.5%)	
T-category (*n* = 71)	I	14	5 (35.71%)	9 (64.29%)	0.82
	II	19	7 (36.84%)	12 (63.16%)	
	III	25	7 (28%)	18 (72%)	
	IV	1	0	1 (100%)	
N-category (*n* = 61)	0	27	8 (29.63%)	19 (70.37%)	0.82
	I	34	11 (32.35%)	23 (67.65%)	
M-category (*n* = 73)	0	53	16 (30.19%)	37 (69.81%)	0.68
	I	8	3 (37.5%)	5 (62.5%)	
Neo-adjuvant therapy	Yes	25	7 (28%)	18 (72%)	0.66
	No	36	12 (33.33%)	24 (66.67%)	
Relapse	Yes	28	8 (28.57%)	20 (71.43%)	0.69
	No	33	11 (33.33%)	22 (66.67%)	
Tumor type (histological) (*n* = 73)	Adenocarcinoma SCC	29	7 (24.14%)	22 (75.86%)	0.34
		31	11 (35.48%)	20 (64.52%)	

**Table 5:** Correlation between CK20 mRNA detection in blood and/or bone marrow of esophageal carcinoma patients and clinical parameters.

Variable		Detection of CK20 mRNA in Blood and/or Bone Marrow (*n* = 77)			Correlation χ^2^-Test (*p*-Value)
		Total	Positive (%)	Negative (%)	
Gender	Female	13	8 (61.54%)	5 (38.46%)	0.448
	Male	64	32 (50%)	32 (50%)	
UICC-stage (*n* = 70)	I	16	7 (43.75%)	9 (56.25%)	0.038
	II	28	18 (64.29%)	10 (35.71%)	
	III	19	6 (31.58%)	13 (68.52%)	
	IV	10	8 (80%)	2 (20%)	
T-category (*n* = 71)	I	18	9 (50%)	9 (50%)	0.598
	II	23	14 (60.87%)	9 (39.13%)	
	III	32	16 (50%)	16 (50%)	
	IV	1	0	1 (100%)	
N-category (*n* = 77)	0	34	19 (55.88%)	15 (44.12%)	0.539
	I	43	21 (48.84%)	22 (51.16%)	
M-category (*n* = 73)	0	66	32 (48.48%)	34 (51.52%)	0.063
	I	10	8 (80%)	2 (20%)	
Neo-adjuvant therapy	Yes	32	17 (53.13%)	15 (46.87%)	0.862
	No	45	23 (51.11%)	22 (48.89%)	
Relapse	Yes	36	19 (52.78%)	17 (47.22%)	0.891
	No	41	21 (51.22%)	20 (48.78%)	
Tumor type (histological) (*n* = 73)	Adenocarcinoma SCC	39	16 (41%)	23 (59%)	0.065
		37	23 (62.16%)	14 (37.84%)	

**Table 6:** Multivariate Cox regression analysis of independent factors influencing overall, tumor-specific and relapse-free survival of esophageal carcinoma patients.

Variables	*p*-Value (Univariate)	*p*-Value (Multivariate)	Hazard Quotient
Overall survival			
CK20 detection in BM	0.029	0.006	2.529
N-category	<0.001	0.357	1.53
UICC stage-group	<0.001	<0.001	3.128
Tumor-specific survival			
CK20 detection in BM	0.048	0.013	2.37
N-category	<0.001	0.548	1.31
UICC stage-group	<0.001	0.002	2.873
Relapse free survival			
N-category	<0.001	0.258	1.646
UICC stage-group	<0.001	0.001	2.913

BM = bone marrow.

## Abbreviations

AEG: adenocarcinomas of the esophagogastric junction; BM: bone marrow; BMB-CCC: Biomaterial bank Comprehensive Cancer Center; CDK: cyclin-dependent kinases; cDNA: complementary deoxyribonucleic acid; CK20: Cytokeratin-20; CTC: circulating tumor cells; DFS: disease-free survival; dNTP: deoxynucleotide triphosphate; DTC: disseminated tumor cells; DTT: Dithiothreitol; EC: esophageal cancer; EDTA: ethylenediaminetetraacetic acid; EMT: epithelial to mesenchymal transition; EpCAM: epithelial cell adhesion molecule; HER2: human epidermal growth factor receptor 2; HR: Hazard Ratio; LQ: likelihood quotient; MET: mesenchymal-epithelial-transition; mRNA: messenger ribonucleic acid; OS: overall survival; NRF2: nuclear factor erythroid 2—related factor 2; RT-PCR: Reverse transcription polymerase chain reaction; RNA: ribonucleic acid; PARP: Poly-Adenosindiphosphat-Ribose-Polymerase; PBS: phosphate buffer saline; PBGD: porphobilinogen deaminase; PD-L1: programmed death-ligand 1; SCC: Squamous cell carcinoma; UICC: Union for International Cancer Control.
